# Swallowing muscle mass contributes to post‐stroke dysphagia in ischemic stroke patients undergoing mechanical thrombectomy

**DOI:** 10.1002/jcsm.13512

**Published:** 2024-06-18

**Authors:** João Pinho, Tareq Meyer, Beate Schumann‐Werner, Johanna Becker, Simone Tauber, Omid Nikoubashman, Martin Wiesmann, Jörg B. Schulz, Cornelius J. Werner, Arno Reich

**Affiliations:** ^1^ Department of Neurology University Hospital RWTH Aachen Germany; ^2^ Department of Neurology and Geriatrics Johanniter Hospital Stendal GmbH Stendal Germany; ^3^ Institute of Cognitive Neurology and Dementia Research Otto Von Guericke University Magdeburg Germany; ^4^ Department of Diagnostic and Interventional Neuroradiology University Hospital RWTH Aachen Germany; ^5^ JARA‐BRAIN Institute Molecular Neuroscience and Neuroimaging Forschungszentrum Jülich GmbH and RWTH Aachen University Aachen Germany

**Keywords:** Dysphagia, Fiberoptic endoscopic evaluation of swallowing, Ischaemic stroke, Sarcopenia

## Abstract

**Background:**

Neurogenic dysphagia is a frequent complication of stroke and is associated with aspiration pneumonia and poor outcomes. Although ischaemic lesion location and size are major determinants of the presence and severity of post‐stroke dysphagia, little is known about the contribution of other acute stroke‐unrelated factors. We aimed to analyse the impact of swallowing and non‐swallowing muscles measurements on swallowing function after large vessel occlusion stroke.

**Methods:**

This retrospective study was based on a prospective registry of consecutive ischaemic stroke patients. Patients who underwent mechanical thrombectomy between July 2021 and June 2022 and received a flexible endoscopic evaluation of swallowing (FEES) within 5 days after admission were included. Demographic, anthropometric, clinical, and imaging data were collected from the registry. The cross‐sectional areas (CSA) of selected swallowing muscles (as a surrogate marker for swallowing muscle mass) and of cervical non‐swallowing muscles were measured in computed tomography. Skeletal muscle index (SMI) was calculated and used as a surrogate marker for whole body muscle mass. FEES parameters, namely, Functional Oral Intake Scale (FOIS, as a surrogate marker for dysphagia presence and severity), penetration aspiration scale, and the presence of moderate‐to‐severe pharyngeal residues were collected from the clinical records. Univariate and multivariate ordinal and logistic regression analyses were performed to analyse if total CSA of swallowing muscles and SMI were associated with FEES parameters.

**Results:**

The final study population consisted of 137 patients, 59 were female (43.1%), median age was 74 years (interquartile range 62–83), median baseline National Institutes of Health Stroke Scale score was 12 (interquartile range 7–16), 16 patients had a vertebrobasilar occlusion (11.7%), and successful recanalization was achieved in 127 patients (92.7%). Both total CSA of swallowing muscles and SMI were significantly correlated with age (rho = −0.391, *P* < 0.001 and rho = −0.525, *P* < 0.001, respectively). Total CSA of the swallowing muscles was independently associated with FOIS (common adjusted odds ratio = 1.08, 95% confidence interval = 1.01–1.16, *P* = 0.029), and with the presence of moderate‐to‐severe pharyngeal residues for puree consistencies (adjusted odds ratio = 0.90, 95% confidence interval = 0.81–0.99, *P* = 0.036). We found no independent association of SMI with any of the FEES parameters.

**Conclusions:**

Baseline swallowing muscle mass contributes to the pathophysiology of post‐stroke dysphagia. Decreasing swallowing muscle mass is independently associated with increasing severity of early post‐stroke dysphagia and with increased likelihood of moderate‐to‐severe pharyngeal residues.

## Introduction

Dysphagia is a well‐known and frequent manifestation of ischaemic stroke and is associated with increased risk of stroke‐associated pneumonia, increased risk of mortality, higher likelihood of poor functional outcomes, worse health related quality‐of‐life, and malnutrition.^1^ Early identification of dysphagia after stroke using screening and diagnostic tests is recommended, because it allows to implement adequate feeding recommendations and plan swallowing therapy and compensation strategies in order to prevent aspiration and other dysphagia‐associated complications.^2^

Although ischaemic lesions in several supratentorial brain regions were shown to be significantly associated with increased risk of aspiration,^3^ ischaemic lesions involving the brainstem, and particularly the lateral medulla oblongata, can classically cause oropharyngeal dysphagia.^4^ Nevertheless, the pathophysiological mechanisms of post‐stroke dysphagia are complex and probably not solely explained by central nervous system ischaemic lesion size and location. In addition to possible neurological and non‐neurological co‐morbidities, which may contribute to swallowing dysfunction and aspiration risk in ischaemic stroke patients, there are also age‐related physiological processes, which may play a role in oropharyngeal dysphagia in these patients.^5^

Mechanisms thought to be involved in the so‐called primary presbyphagia (age‐related impairment of swallowing mechanisms) include weakness of the masticatory and swallowing muscles due to sarcopenia, decreased elasticity of the pharyngeal and laryngeal connective tissue, reduced salivary flow rates, delayed triggering of the swallow reflex, as well as impaired pharyngeal sensory function and oesophageal peristalsis.^6^, ^7^ Although it has been known for a long time that weakness of swallowing muscles develops in older people,^8^ the concept of sarcopenic dysphagia emerged just recently^9^ and is defined as dysphagia associated with both generalized sarcopenia and sarcopenia of the swallowing muscles after other causes for relevant swallowing impairment have been excluded.^10^ The relationship between sarcopenia and dysphagia is complex, because sarcopenia is also a marker for frailty, malnutrition and increased co‐morbidities.^11^

We hypothesized that sarcopenia may play a role in the severity of post‐stroke dysphagia and aimed to analyse the relevance of swallowing and non‐swallowing muscles measurements on swallowing function after acute ischaemic stroke in patients who underwent mechanical thrombectomy.

## Methods

This single‐centre retrospective study is based on a prospective registry of consecutive ischaemic stroke patients undergoing mechanical thrombectomy during a 1‐year period (July 2021–June 2022). Beginning in July 2021, we changed our routine clinical care in respect to swallowing assessment: We aim to perform flexible endoscopic evaluation of swallowing (FEES) in all ischaemic stroke patients undergoing mechanical thrombectomy within 5 days of admission regardless of the results of initial dysphagia screening, unless there were contraindications, the patient was receiving palliative treatment or the patient did not consent to the exam. In the current study, we included only patients with large vessel occlusion who underwent mechanical thrombectomy and received FEES during the first 5 days after hospital admission. This decision was based on the fact that, in our department, stroke patients who do not receive mechanical thrombectomy do not systematically receive swallowing assessment by FEES; therefore, we tried to reduce selection bias by excluding these patients from our study. Patients who received FEES later than 5 days after admission were excluded from this study to minimize the impact of possible weight and muscle mass loss during hospital admission. Baseline demographic (age and sex), anthropometric data [weight, height, and body mass index (BMI)], co‐morbidities (vascular risk factors and atrial fibrillation), baseline clinical and imaging stroke severity [National Institutes of Health Stroke Scale (NIHSS), Alberta Stroke Program Early Computed Tomography Score (ASPECTS) or posterior circulation ASPECTS (pcASPECTS)], arterial territory of the occlusion, treatment with intravenous thrombolysis and achievement of successful recanalization [defined as a revised thrombolysis in cerebral infarction (TICI) score of 2b, 2c or 3^12^] were collected from the registry and individual patient records. Pre‐stroke medical diagnoses were coded using the International Classification of Diseases 10th revision (ICD‐10). Using pre‐stroke ICD‐10 codes, we calculated the pre‐stroke Hospital Frailty Risk Score (HFRS), which is a validated marker for frailty risk in patients admitted to hospital.^13^ HFRS scores <5 represent low frailty risk, scores between 5–15 represent intermediate frailty risk, and scores >15 represent high frailty risk. We reviewed follow‐up computed tomography (*n* = 53) and magnetic resonance (*n* = 84) imaging for each patient, to define if there was an acute infarct involving the brainstem or, specifically, the medulla oblongata. The presence of bilateral acute or chronic fronto‐opercular lesions was also assessed.

### Flexible endoscopic evaluation of swallowing

FEES was performed according to a standardized protocol by experienced and certified speech and language therapists accredited according to the accreditation program of the German Society for Neurology.^14^ All examinations and reports were medically supervised. If patients were initially intubated and ventilated, we avoided performing FEES within the first 24 h after extubation to avoid possible residual sedation‐related reduced vigilance and possible unstable cardiorespiratory or neurological conditions. FEES was only performed in patients who presented a normal level of vigilance and who were compliant with the examination. Only a minority of patients had already a nasogastric tube inserted at the time of FEES; dietary recommendations for non‐oral feeding were almost always decided after the performance of FEES. The local FEES protocol includes an initial evaluation of the anatomy and function (observation at rest, elevation of the soft palate, phonation, and saliva management), followed by consecutive oral intake of different food consistencies, starting with puree consistency and followed by thin liquid consistency and solid consistency. Mixed and thick liquid consistencies are tested at the discretion of the examiner.

The penetration aspiration scale (PAS) was systematically documented in our FEES reports. It describes the occurrence of airway invasion events (penetration and aspiration) and associated response for each tested consistency as the primary marker for swallowing safety. PAS ranges from normal findings (1), to penetration scores (2–5), to aspiration scores (6–8).^15^ Steele and Grace‐Martin proposed a modified 4‐level PAS (4L‐PAS) to overcome limitations related to ordinal and interval qualities of PAS.^16^ An increasing 4L‐PAS reflects an increasing severity of penetration or aspiration and an increasing severity of impaired response to penetration or aspiration. We derived 4L‐PAS from PAS documented in individual clinical records and used it as surrogate marker for the presence and severity of penetration and aspiration. In order to capture swallowing efficiency, residues in the pyriform sinuses and in the valleculae for each of the tested consistencies were systematically documented in the FEES report using the Yale Pharyngeal Residue Severity Rating Scale (YPRSRS).^17^ A score of 4 of 5 in the YPRSRS is defined as moderate to severe residues. We used predominance of residues in the pyriform sinus as an indirect marker for upper oesophageal muscle opening dysfunction, and we defined it as an YPRSRS for the pyriform sinus higher than YPRSRS for the valleculae, for each consistency. We used predominance of residues in the valleculae as an indirect marker for reduced pharyngeal contractility, and we defined it as an YPRSRS for the valleculae higher than YPRSRS for the pyriform sinus, for each consistency. The presence of predominance of residues in the pyriform sinus was considered present if this YPRSRS difference was observed for at least one consistency. Finally, recommendations for food intake using the Functional Oral Intake Scale (FOIS) were systematically documented and based on the overall judgement of SLT and clinicians according to the findings in FEES. We used the FOIS as a correlate for the presence and severity of swallowing impairment.^18^ FOIS is an ordinal scale, which ranges from non‐oral feedings scores (1–3), to adjusted or restricted oral feeding scores (4–6), up to a maximal score for oral feeding with no restrictions (7). Individual clinical records of all patients were reviewed to collect PAS, FOIS and the presence of moderate‐to‐severe pharyngeal residues according to the YPRSRS from the FEES report.

### Imaging protocol and muscle measurements

**Figure 1:**
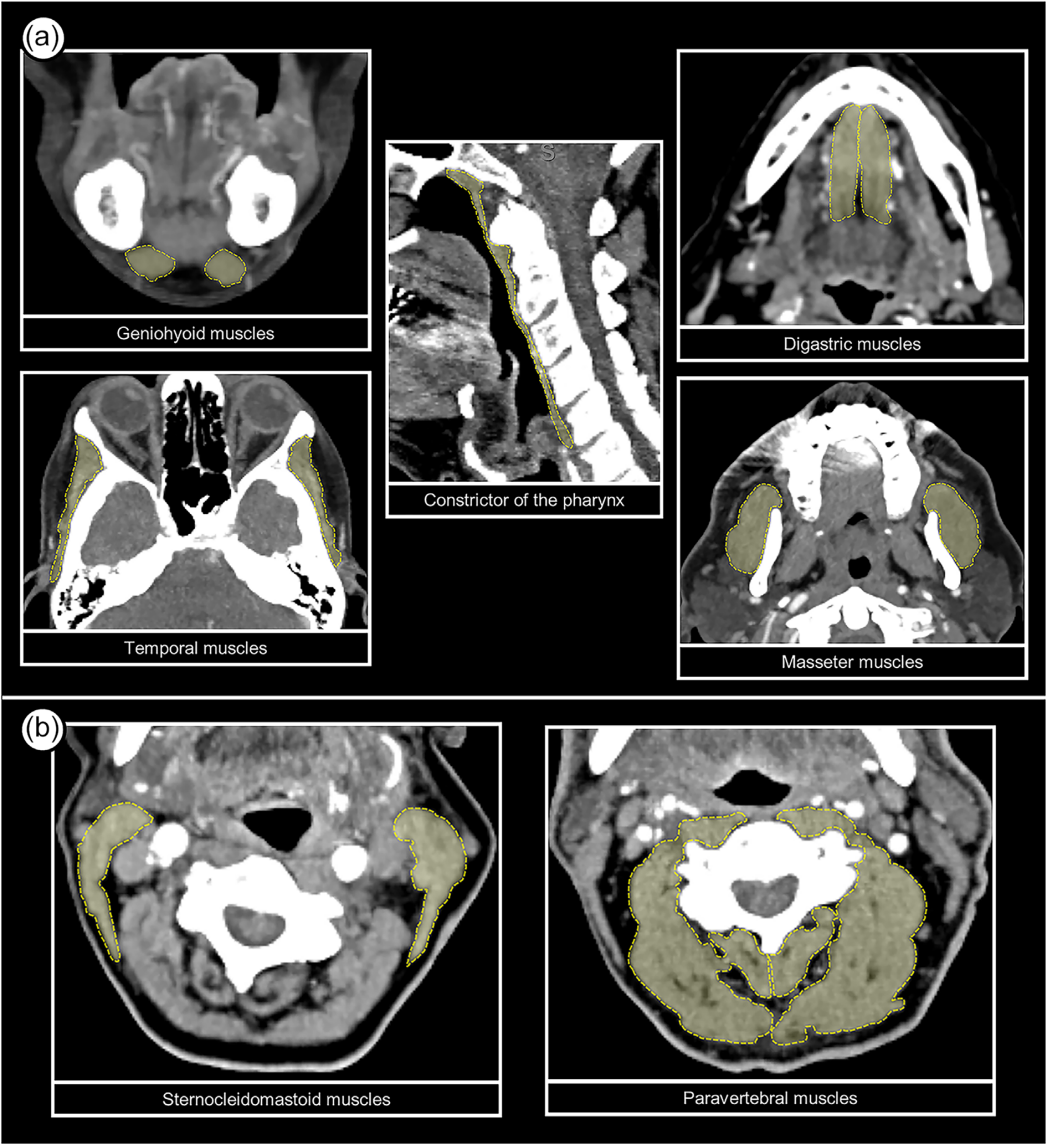
Examples of cross‐sectional area measurements of swallowing muscles (A) and of non‐swallowing cervical muscles used to calculate skeletal muscle index (B).

Computed tomography angiography (CTA) of the supraaortic arteries was performed in all included patients as a part of the stroke emergent care at hospital admission and used to measure the cross‐sectional areas (CSA) of selected muscles. CT scans were performed on a multi‐detector CT scanner (SOMATOM Definition AS, Siemens, Erlangen, Germany) using a collimation of 40 × 0.6 mm with 3 mm maximum intensity projection reformations in an intermediate kernel (B20f) and a reference tube–current–time product of 120 mAs. Cross‐sectional areas (cm^2^) of the following muscles were manually segmented by a senior neuroradiologist (>15 years of experience) using the Picture Archiving and Communication System (PACS): temporal (axial, bilateral, and at the mid orbital level), masseter (axial, bilateral, and at the inferior level of the maxillary bone), geniohyoid (axial, bilateral, and at the mid‐lower level of the mandible body), digastric (coronal, bilateral, and immediately anterior to the anterior wall of the sphenoidal sinus), constrictor of the pharynx (sagittal and midline), sternocleidomastoid (axial, bilateral, and at the level of C3), and paravertebral muscle mass (axial, bilateral, and at the level of C3). Examples of measurements of each muscle can be found in *Figure* 1. For the evaluation of the intra‐rater reliability of CT‐based muscle measurements, the same rater who performed the initial CSA measurements performed again the same measurements in 30 randomly selected patients from the study population. For the evaluation of the inter‐rater reliability, a senior vascular neurologist (>15 years of experience) performed the same measurements in the same 30 randomly selected patients. We calculated intraclass correlation coefficients (ICC) and respective 95% confidence intervals (95% CI) for each muscle measurement. All CSA measurements were performed blinded for the presence of dysphagia and for dietary recommendations. Total CSA of swallowing muscles was calculated as the sum of the CSA of all swallowing muscles, which were measured (temporal, masseter, geniohyoid, digastric, and constrictor of the pharynx) and used as a surrogate marker for swallowing muscle mass. The skeletal muscle index (SMI, cm^2^/m^2^), which is a surrogate measure of whole‐body muscle mass, was derived from the CSA of sternocleidomastoid and paravertebral muscles at the level of C3, as proposed by Zwart et al.^19^

### Statistical analyses

Descriptive statistics of the baseline variables were performed. Data were presented as number (%) or median [interquartile range (IQR)] as adequate. Spearman correlations were performed to analyse correlations between age, total CSA of swallowing muscles and SMI. We divided our study population in patients with recommendation to no dietary restriction (FOIS = 7) and in patients with recommendation to dietary restrictions (FOIS 6–7) according to FEES, and compared baseline characteristics of both groups by using chi‐square tests and Mann–Whitney tests, as adequate. Univariate and multivariate ordinal regression analyses were conducted to analyse if total CSA of swallowing muscles and SMI were predictors of the severity of swallowing impairment (defined by FOIS as an ordinal variable ranging from 1 to 7) and of swallowing safety (defined by the worst 4L‐PAS among all consistencies tested, defined as an ordinal variable ranging from 1 to 4). Univariate and multivariate binary logistic regression analyses were conducted to analyse if total CSA of swallowing muscles and SMI were predictors of swallowing efficiency (as defined by the severity of pharyngeal residues for each of the tested consistencies). Odds ratios (OR), common OR (cOR), 95% CI, and associated *P*‐values are presented. Variables selected to be included as covariates in the multivariate regression models were variables, which are known to influence global muscle mass (age, sex, BMI, and frailty as measured by pre‐stroke HFRS) and variables known to influence the presence and severity of early post‐stroke dysphagia (baseline NIHSS, baseline ASPECTS/pcASPECTS, vertebrobasilar occlusion, and successful recanalization). Age, sex, and BMI were not included as a covariate for analysis of SMI, because age, sex, height, and weight are already taken into consideration in the calculation of this index. The level of significance was set at an alpha value of 0.05.

## Results

Among a total of 247 patients with acute ischaemic stroke due to large vessel occlusion who underwent mechanical thrombectomy during the study period, a total of 137 patients received FEES within 5 days after hospital admission. The main reasons for not receiving FEES within 5 days after admission were no extubation during the first 5 days after mechanical thrombectomy (*n* = 38), death or institution of palliative care within 5 days of admission (*n* = 29), interhospital transfer within 5 days of admission before FEES could be performed (*n* = 20), incompliance or reduced vigilance (*n* = 12), and other reasons (*n* = 11).

The final study population consisted of 137 patients with a median age of 74 years (IQR = 62–83), 59 were female (43.1%), median BMI was 25.8 (IQR = 23.9–29.2), median pre‐stroke HFRS was 0 and only 8% of patients had HFRS scores greater than 5. Median baseline NIHSS was 12 (IQR = 7–16) and median baseline ASPECTS/pcASPECTS was 9 (IQR = 8–10). Sixteen patients had a vertebrobasilar occlusion (11.7%), only 5 patients had brainstem infarct on follow‐up imaging and no patient had an infarct on the medulla oblongata. No patient with lesions or impairment of central pattern generators for swallowing was included. Three patients presented bilateral acute and/or chronic fronto‐opercular lesions, and two of these patients did not present any dysphagia. Sixty‐four patients received intravenous thrombolysis (46.7%) and successful recanalization was achieved in 127 patients (92.7%). Among all included patients, 23 (16.8%) were dependent on nasogastric tube for feeding (FOIS 1–3), 79 (57.7%) were recommended to receive a restricted oral feeding (FOIS 4–6), and 35 patients (25.5%) were recommended to have a total oral diet with no restrictions (FOIS 7). A score of 4 or 5 in the YPRSRS, indicative of moderate to severe pharyngeal residues, was found in 60 patients (43.8%): in 27% of patients for puree consistency, in 14% of patients for thin liquid consistency and in 23% of patients for solid consistency. Predominance of residues in the valleculae was observed in 89 patients (65.0%), and predominance of residues in the pyriform sinus was observed in only five patients (3.6%). Patients with no dietary restrictions based on FEES (FOIS = 7) were younger (median 64 vs. 76 years, *P* = 0.004), had higher BMI (median 28.1 vs. 25.4, *P* = 0.013), had lower pre‐stroke HFRS (*P* = 0.007), had lower baseline NIHSS (median 7 vs. 13, *P* < 0.001), had lower baseline ASPECTS/pcASPECTS (median 10 vs. 9, *P* = 0.009), and were more frequently treated with intravenous thrombolysis (62.9% vs. 41.2%, *P* = 0.027) (*Table* 1).

**Table 1:** Comparison of the baseline characteristics of the study population according to the recommendation of dietary restrictions (based on flexible endoscopic evaluation of swallowing)

	Dietary restrictions recommended (*n* = 102)	No dietary restrictions recommended (*n* = 35)	*P*
Age (years)	76 (65–84)	64 (57–78)	0.004
Female sex	45 (44.1)	14 (40.0)	0.671
Body mass index	25.4 (23.7–27.8)	28.1 (24.0–30.7)	0.013
Pre‐stroke HFRS	0 (0–2)	0 (0–1)	0.007
Arterial hypertension	85 (83.3)	26 (74.3)	0.239
Diabetes mellitus	29 (28.4)	15 (42.9)	0.115
Dyslipidaemia	57 (55.9)	17 (48.6)	0.454
Active smoking	20 (19.8)	8 (22.9)	0.700
Baseline NIHSS	13 (8–16)	7 (4–13)	<0.001
Baseline ASPECTS/pcASPECTS	9 (8–10)	10 (9–10)	0.009
Vertebrobasilar territory occlusion	12 (11.8)	4 (11.4)	0.957
Brainstem infarct	5 (4.9)	0	0.182
Intravenous thrombolysis	42 (41.2)	22 (62.9)	0.027
Successful revascularization	94 (92.2)	33 (94.3)	0.676

Results are present as number (%) or median (P25–P75).

HFRS, Hospital Frailty Risk Score; NIHSS, National Institutes of Health Stroke Scale, ASPECTS, Alberta Stroke Program Early Computed Tomography Score; pcASPECTS, posterior circulation Alberta Stroke Program Early Computed Tomography Score.

Intra‐rater agreement for CSA muscle measurements was excellent (ICC > 0.90) for all muscles except for geniohyoid and digastric muscles, which were good (ICC = 0.73 and 0.83, respectively; *Table* S1). Inter‐rater agreement for CSA muscle measurements was excellent (ICC >0.90) for all muscle except for geniohyoid, digastric and pharyngeal constrictor muscle, which were good (ICC = 0.79, 0.81 and 0.82, respectively; *Table* S1).

**Figure 2:**
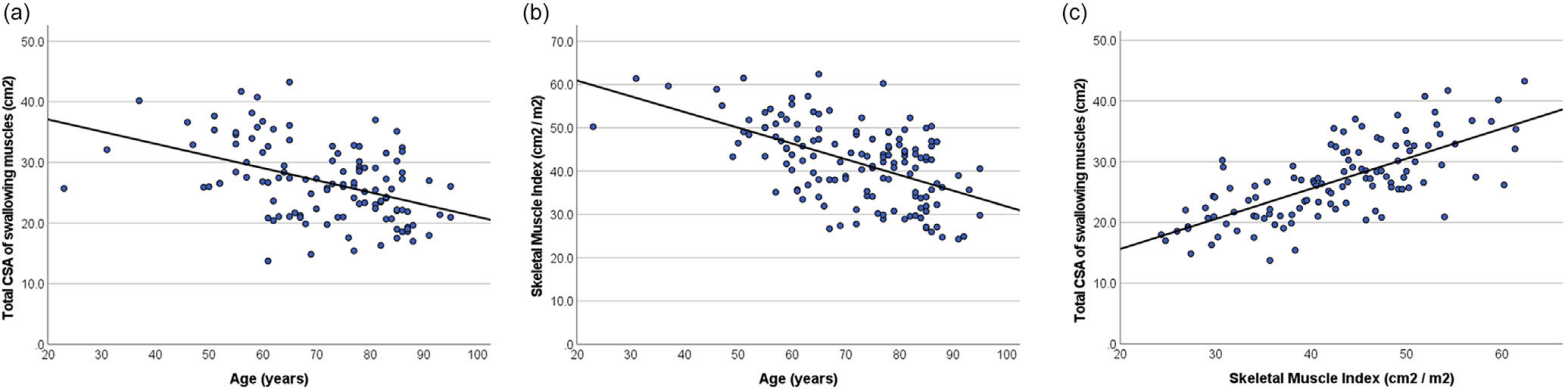
Scatterplot describing the distribution of total cross‐sectional area (CSA) of swallowing muscles (A) and skeletal muscle index (B) according to age, and the distribution of total CSA of swallowing muscles according to the skeletal muscle index (C).

Age was significantly negatively correlated with both total CSA of swallowing muscles (rho = −0.391, *P* < 0.001) and SMI (rho = −0.525, *P* < 0.001). Distribution of total CSA of swallowing muscles and SMI according to age is depicted in Figure 2A and 2B, respectively. The estimation of whole‐body muscle mass, measured by SMI, was also strongly correlated with total CSA of swallowing muscles (rho = 0.705, *P* < 0.001) (*Figure* 2C). Pre‐stroke HFRS was not significantly correlated with total CSA of swallowing muscles (rho = −0.161, *P* = 0.060) or with SMI (rho = −0.118, *P* = 0.168). Total CSA of swallowing muscles and SMI were not differently distributed in patients with or without predominance of residues in the pyriform sinus (*P* = 0.574 and *P* = 0.688, respectively).

The analyses for predictors of the severity of swallowing impairment revealed that total CSA of swallowing muscles was a predictor of FOIS in the univariate ordinal regression analyses. This association persisted after adjustment for relevant confounders in the multivariate ordinal regression analysis (*Table* 2). An increasing total CSA of swallowing muscles was independently associated with increasing FOIS (cOR per 1 cm^2^ increase = 1.08, 95% CI = 1.01–1.16; total CSA of swallowing muscles and SMI were not differently distributed in patients with or without predominance of residues in the pyriform sinus [*P* = 0.574 and *P* = 0.688, respectively), but were *P* = 0.029]. SMI was not associated with FOIS in the univariate or multivariate ordinal regression analyses (*Table* 2). Concerning prediction of swallowing safety, we found no independent association of total CSA of the swallowing muscles or of the SMI with the worst 4L‐PAS (*Table* 3). Total CSA of the swallowing muscles was independently associated with moderate‐to‐severe pharyngeal residues for puree consistencies [OR = 0.90, 95% CI = 0.81–0.99, total CSA of swallowing muscles and SMI were not differently distributed in patients with or without predominance of residues in the pyriform sinus (*P* = 0.574 and *P* = 0.688, respectively), but were *P* = 0.036], and there was a non‐significant trend for it to be associated with moderate‐to‐severe residues for thin liquid consistencies [OR = 0.88, 95% CI = 0.77–1.00, total CSA of swallowing muscles and SMI were not differently distributed in patients with or without predominance of residues in the pyriform sinus (*P* = 0.574 and *P* = 0.688, respectively), but were *P* = 0.053] (*Table* 4). There was no association of total CSA of the swallowing muscles with moderate‐to‐severe residues for solid consistencies in the 113 patients who were tested for solid consistencies (*Table* 4). We found no association of SMI with the presence of moderate‐to‐severe pharyngeal residues for any consistency.

**Table 2:** Univariate and multivariate ordinal regression analyses for prediction of severity of swallowing impairment using functional Oral intake scale as the dependent variable

	Common odds ratio (95% confidence interval)	*P*
Total CSA of the swallowing muscles (per 1 cm^2^ increase)		
Unadjusted	1.08 (1.03–1.15)	0.002
Model 1	1.09 (1.02–1.17)	0.018
Model 2	1.08 (1.01–1.16)	0.029
Skeletal muscle index (per 1 cm^2^/m^2^ increase)		
Unadjusted	0.99 (0.94–1.04)	0.612
Model 3	1.02 (0.99–1.06)	0.581

Model 1: adjusted for age, sex, body mass index and Hospital Frailty Risk Score. Model 2: adjusted for age, sex, body mass index, Hospital Frailty Risk Score, baseline National Institutes of Health Stroke Scale, baseline Alberta Stroke Program Early Computed Tomography Score/posterior circulation Alberta Stroke Program Early Computed Tomography Score, vertebrobasilar occlusion, successful recanalization. Model 3: adjusted for Hospital Frailty Risk Score, baseline National Institutes of Health Stroke Scale, baseline Alberta Stroke Program Early Computed Tomography Score/posterior circulation Alberta Stroke Program Early Computed Tomography Score, vertebrobasilar occlusion, successful recanalization.

CSA, cross‐sectional area.

**Table 3:** Univariate and multivariate ordinal regression analyses for prediction of swallowing safety using the worst score on the modified 4‐level penetration aspiration scale as the dependent variable

	Common odds ratio (95% confidence interval)	*P*
Total CSA of the swallowing muscles (per 1 cm^2^ increase)		
Total CSA of the swallowing muscles, unadjusted	0.95 (0.90–1.00)	0.062
Total CSA of the swallowing muscles, Model 1	0.99 (0.92–1.06)	0.819
Total CSA of the swallowing muscles, Model 2	1.01 (0.93–1.08)	0.870
Skeletal muscle index (per 1 cm^2^/m^2^ increase)		
Unadjusted	0.96 (0.93–1.00)	0.049
Model 3	0.97 (0.94–1.01)	0.162

Model 1: adjusted for age, sex, body mass index and Hospital Frailty Risk Score. Model 2: adjusted for age, sex, body mass index, Hospital Frailty Risk Score, baseline National Institutes of Health Stroke Scale, baseline Alberta Stroke Program Early Computed Tomography Score/posterior circulation Alberta Stroke Program Early Computed Tomography Score, vertebrobasilar occlusion, successful recanalization. Model 3: adjusted for Hospital Frailty Risk Score, baseline National Institutes of Health Stroke Scale, baseline Alberta Stroke Program Early Computed Tomography Score/posterior circulation Alberta Stroke Program Early Computed Tomography Score, vertebrobasilar occlusion, successful recanalization.

CSA, cross‐sectional area.

**Table 4:** Univariate and multivariate binary logistic regression analyses for prediction of swallowing efficiency using moderate‐to‐severe pharyngeal residues (based on the Yale Pharyngeal Residue Severity Rating Scale) for each tested consistency as the dependent variable

	Moderate‐to‐severe pharyngeal residues ‐ thin liquid consistency		Moderate‐to‐severe pharyngeal residues ‐ puree consistency		Moderate‐to‐severe pharyngeal residues ‐ solid consistency^a^	
	OR (95% CI)	*P*	OR (95% CI)	*P*	OR (95% CI)	*P*
Total CSA of the swallowing muscles (per 1 cm^2^ increase)						
Unadjusted	0.96 (0.88 to 1.04)	0.311	0.93 (0.87 to 1.00)	0.044	0.97 (0.91 to 1.04)	0.454
Model 1	0.88 (0.78 to 1.00)	0.047	0.89 (0.81 to 0.99)	0.026	0.99 (0.90 to 1.01)	0.763
Model 2	0.88 (0.77 to 1.00)	0.053	0.90 (0.81 to 0.99)	0.036	0.98 (0.89 to 1.08)	0.719
Skeletal muscle index (per 1 cm^2^/m^2^ increase)						
Unadjusted	0.99 (0.94 to 1.05)	0.759	0.98 (0.94 to 1.02)	0.278	0.99 (0.95 to 1.04)	0.835
Model 3	1.00 (0.94 to 1.06)	0.975	0.98 (0.94 to 1.03)	0.446	1.00 (0.95 to 1.05)	0.855

Model 1: adjusted for age, sex, body mass index and Hospital Frailty Risk Score. Model 2: adjusted for age, sex, body mass index, Hospital Frailty Risk Score, baseline National Institutes of Health Stroke Scale, baseline Alberta Stroke Program Early Computed Tomography Score/posterior circulation Alberta Stroke Program Early Computed Tomography Score, vertebrobasilar occlusion, successful recanalization. Model 3: adjusted for Hospital Frailty Risk Score, baseline National Institutes of Health Stroke Scale, baseline Alberta Stroke Program Early Computed Tomography Score/posterior circulation Alberta Stroke Program Early Computed Tomography Score, vertebrobasilar occlusion, successful recanalization.

95% CI, 95% confidence interval; CSA, cross‐sectional area; OR, odds ratio.

^a^ Solid consistencies tested in 113 patients.

## Discussion

The main finding of our study is that a surrogate marker of swallowing muscle mass is independently associated with severity of early dysphagia in acute large vessel occlusion stroke patients who undergo mechanical thrombectomy. Of note, decreasing swallowing muscle mass is associated with increased severity of dysphagia, which is physiologically plausible. One of the main implications of these results is that factors other than stroke severity and ischaemic lesion location may also play an important role in the pathophysiology of post‐stroke dysphagia. This aspect is often overlooked in studies assessing clinical and imaging stroke‐associated predictors of dysphagia, and may lead to bias in the clinical judgement of dysphagia in patients with small ischaemic lesions in locations not typically associated with dysphagia. Our study supports the hypothesis that pre‐existing swallowing muscle changes may increase the likelihood of overt dysphagia by impairing the ability for compensating disease‐related swallowing dysfunction.^5^

Increasing age is known to be a risk factor for post‐stroke dysphagia,^20^ and it is also associated with sarcopenia and changes of the swallowing function, which may be disease‐independent.^21^, ^22^ Additional age‐related changes of swallowing include reduced saliva production,^23^ impaired pharyngeal sensory function,^7^ and delayed swallowing reflex.^24^ Additionally, different electromyographical activation patterns of swallowing muscles were found in healthy older adults in comparison to healthy younger adults,^25^ which suggests the existence of mechanisms of adaptation to swallowing muscle loss in older adults. Disentangling the reasons for the association between increasing age and post‐stroke dysphagia may be difficult, because older patients with ischaemic stroke tend to have more severe strokes and present more frequently with reduced vigilance.^26^ Although increasing age is associated with higher frequency of sarcopenia, many other factors that are not necessarily related to age may cause loss of muscle mass and loss of muscle strength, such as low protein and energy intake, gastrointestinal diseases causing malabsorption, physical inactivity, cardiorespiratory, renal and hepatic diseases, metabolic disorders, endocrine disorders, and cancer.^27^ In our study, swallowing muscle mass was able to predict severity of swallowing impairment independently of age. We tried to capture the possible influence of factors that are not necessarily associated with age on both muscle mass and swallowing function by measuring frailty risk in our study population. Frailty is a disorder of multiple physiological systems resulting in decreased physiological reserve when facing insult or stress associated with medical conditions. One of the physiological systems that may be involved is the muscular system, with loss of muscle mass and loss of muscle strength.^28^ Frailty in stroke patients leads to worse functional outcomes,^29^ and this effect is partially mediated through sarcopenia.^30^ However, the prediction of swallowing impairment by using swallowing muscle mass in our study population was also independent of frailty risk, which suggests the presence of several other specific contributors for loss of swallowing muscle mass. We recognize that the distribution of frailty risk measured by pre‐stroke HFRS in our study population was significantly skewed to lower frailty risk scores, which limits the study of the complex relationship between post‐stroke dysphagia, pre‐stroke sarcopenia, and pre‐stroke frailty. Other frailty measures, such as Clinical Frailty Scale, might yield different results but were not available for this study.

Our study confirms the results of a previous study by Sporns et al., which showed that the volume of swallowing muscles predicted the severity of post‐stroke dysphagia measured by FEES.^31^ In this study, this association was not independent from age and clinical stroke severity, and other important confounders, such as sex and BMI, were not accounted for. Another recent smaller study showed that temporal muscle thickness in ischaemic stroke and intracerebral haemorrhage patients was independently associated with severity of dysphagia defined by FOIS.^32^ However, relevant limitations of this study include failure to account for anthropometric characteristics of the patients and absent instrumental method to diagnose dysphagia and to define food intake recommendations.

Although total CSA of the swallowing muscles was not a predictor of the presence and severity of penetration or aspiration, it was independently associated with the presence of moderate‐to‐severe pharyngeal residues for puree consistencies. A marginal non‐significant association was also found for moderate‐to‐severe pharyngeal residues for liquid consistencies. It is known that patients with myositis, muscular dystrophies and disorders of the neuromuscular junction, which cause weakness of the swallowing muscles, often present a pattern of dysphagia that predominantly causes impairment of the pharyngeal phase and increased pharyngeal residues.^33^ This is probably explained by reduced elevation of the hyolaryngeal complex, reduced opening of the upper oesophageal sphincter and/or reduced pharyngeal constriction.^34^ Our results suggest that the main mechanism by which decreased swallowing muscle mass contributes to early post‐stroke dysphagia is by increasing pharyngeal residues, namely, in the valleculae, which may be explained primarily by the contribution of reduced pharyngeal contractility. This, together with the stroke‐induced disruption of the neuronal network responsible for the control of swallowing, may consequently increase the risk of relevant aspiration and aspiration pneumonia known to occur in ischaemic stroke patients with dysphagia.^1^ Although delayed swallowing reflex and premature bolus spillage were found to be the leading mechanisms in post‐stroke dysphagia in the study by Warnecke et al.,^35^ our data suggest that moderate‐to‐severe pharyngeal residues are also important contributing pathophysiological factors for early dysphagia in this population of patients. Similarly to what has been previously described,^35^ predominance of pharyngeal residues in the pyriform sinus (an indirect marker for upper oesophageal sphincter opening dysfunction) appears to occur infrequently in this population of patients with predominantly anterior circulation strokes, and it was not associated in our study with total CSA of swallowing muscles.

Another important finding of our study is that SMI, a surrogate marker for whole‐body muscle mass, was strongly correlated with total CSA of swallowing muscles but was not associated with the severity of early post‐stroke dysphagia in our study population. This suggests that whole body sarcopenia is less important than the specific sarcopenia of swallowing muscles in the pathophysiology of post‐stroke dysphagia. Although whole body sarcopenia was associated with the presence of dysphagia in several studies^21^ and it may be a surrogate marker for the loss of swallowing muscle mass, our findings suggest that the specific assessment of swallowing muscles may be more relevant for the prediction of dysphagia, even in patients with conditions that can classically cause dysphagia. Early recognition of sarcopenia of the swallowing muscles may help dysphagia risk stratification of ischaemic stroke patients and contribute to a more precise tailoring of the feeding recommendations as well as swallowing training programs. Targeted training of swallowing muscles was indeed shown to improve dysphagia after stroke.^36^ It is also interesting to note that the development of decreased muscle mass and decreased muscle strength after the acute phase of stroke was associated with the occurrence of delayed dysphagia after stroke and could explain worsening of early post‐stroke dysphagia,^37^ but this was not specifically examined in our study.

The main strengths of our study include the availability of a validated instrumental assessment of swallowing, the use of objective scales to characterize not only the severity of dysphagia, but also the presence of penetration, aspiration and pharyngeal residues, and the adjustment of our results for important confounders (viz., BMI, and clinical and imaging stroke severity). Muscle measurements were carried out using imaging at hospital admission, which excludes the effect of the development of muscle loss after stroke due to inactivity or malnutrition.

The main limitations of our study include the relatively small population size, the retrospective nature of the study, absent objective information on pre‐stroke swallowing function and unavailable measurements of the infarct sizes. We opted to measure cross‐sectional areas of muscles. However, volume measurements may be more accurate to define muscle mass. We did not measure muscle densities, which could provide more detailed information concerning sarcopenia and muscle fatty infiltration. We used CT for muscle measurements, because it is the imaging method routinely available in these patients. However, photon‐counting CT or magnetic resonance are methods that are able to provide a better definition of muscles and their structure. Other markers of sarcopenia, such as hand‐grip strength and bioelectrical impedance analysis, were not available for our population of patients. Additionally, only ischaemic stroke patients undergoing mechanical thrombectomy were included in this study, which limits the generalisability of these results to all ischaemic stroke patients. The videofluoroscopic swallowing study is another instrumental dysphagia diagnostic tool that may be more sensitive than FEES for the detection of aspiration, but it is not routinely performed in our centre for evaluation of acute stroke patients, which also represents a limitation of this study.^38^

In conclusion, swallowing muscle mass independently contributes to early post‐stroke dysphagia in ischaemic stroke patients undergoing mechanical thrombectomy. Research identifying stroke‐unrelated factors that contribute to post‐stroke dysphagia is important, because such factors may be therapeutic targets.

## Conflict of interest

SC Tauber has served on the scientific advisory boards of Roche and Merck & Co and has received travel and speaker honoraria from Novartis, Teva, Merck & Co, Roche, and Biogen. The remaining authors report no conflict of interests.

## Supporting information


**Table S1.** Intra‐rater and inter‐rater agreement for cross sectional area muscle measurements in thirty randomly selected patients from the study population.
