# Supplementary material for: Swallowing muscle mass contributes to post‐stroke dysphagia in ischemic stroke patients undergoing mechanical thrombectomy
Source: J Cachexia Sarcopenia Muscle. 2024 Jun 18;15(4):1539–48. doi: 10.1002/jcsm.13512 (PMC11294029; doi:10.1002/jcsm.13512)
Supplement: Supplementary file 1 — Table S1. Intra‐rater and inter‐rater agreement for cross sectional area muscle measurements in thirty randomly selected patients from the study population. [file JCSM-15-1539-s001.docx]

SUPPLEMENTARY MATERIAL

Supplementary Table 1. Intra-rater and inter-rater agreement for cross sectional area muscle measurements in thirty randomly selected patients from the study population.

|  | **Intraclass correlation coefficient**  **(95% confidence interval)** | |
| --- | --- | --- |
|  | **Intra-rater agreement** | **Inter-rater agreement** |
| Geniohyoid muscles | 0.733 (0.439-0.873) | 0.789 (0.557-0.900) |
| Temporal muscles | 0.948 (0.891-0.975) | 0.944 (0.881-0.973) |
| Masseter muscles | 0.966 (0.928-0.984) | 0.946 (0.888-0.975) |
| Digastric muscles | 0.825 (0.631-0.916) | 0.808 (0.596-0.908) |
| Pharyngeal constrictor muscle | 0.939 (0.872-0.971) | 0.817 (0.615-0.913) |
| Sternocleidomastoid muscles | 0.985 (0.969-0.993) | 0.979 (0.956-0.990) |
| Paravertebral muscles | 0.976 (0.950-0.989) | 0.972 (0.941-0.987) |
